# Lower limb electromyographic characteristics and implications of taekwondo roundhouse kick “hit” and “miss” actions

**DOI:** 10.3389/fbioe.2023.1258613

**Published:** 2024-01-26

**Authors:** Jianbo Sun, Yifei Wang, Delong Dong

**Affiliations:** Department of Physical Education, Ludong University, Yantai, China

**Keywords:** taekwondo, EMG, techniques and tactics, sports training, enlightenment

## Abstract

To compare the muscular characteristics of “hit” and “miss” actions in roundhouse kicks among taekwondo athletes, and explore the similarities, differences, and implications for training, motion tests were conducted on ten taekwondo athletes using Noraxon32 and VICON. The results showed no significant differences (*p* > 0.05) in integrated electromyography (EMG) during the initiation and kicking phases between “miss” and “hit” actions. However, during the retraction phase, significant differences (*p* < 0.05) were observed in the left rectus femoris, left peroneus longus, right biceps femoris, right semitendinosus, and right tibialis anterior muscles. The tibialis anterior muscle of the swinging leg was activated first in the “hit” action, while the biceps femoris was activated first in the “miss” action. The supporting-side rectus femoris was activated first in the “hit” action, whereas it was the biceps femoris in the “miss” action. In both techniques, the gluteus maximus was the last muscle to be activated. The “miss” action had a longer cycle, and the duration of muscle work was longer than in the “hit” action. During the retraction phase of the front leg roundhouse kick, the muscles worked more than during the kicking phase, with the erector spinae and tibialis anterior being the core force-producing muscles in both techniques, characterized by high EMG values and long activation times. In the “miss” action, the thigh muscles drove the calf muscles, while the “hit” action exhibited the opposite pattern. “Hit” actions had a faster cycle compared to “miss,” with greater force generation in “miss.” The hip flexors and knee extensors of the kicking leg were the core force-producing muscles during the kicking process, determining the effectiveness and completion of the action.

## 1 Introduction

Taekwondo features several key techniques, including the roundhouse kick, side kick, spinning kick, back kick, inside kick, and outside crescent kick. Moreover, these techniques can be adjusted to target different areas, such as high (head) and mid-section (torso) targets. “Roundhouse kick” is often referred to as the “first leg technique” in Taekwondo. It was the most commonly used technique before the introduction of new rules (Gao et al., 2010; Moenig et al., 2023). The reason for its popularity lies in the fact that the roundhouse kick has a long striking distance and considerable power, making it compatible with various other kicking techniques (Lin et al., 2015; Liu, 2022). In a competitive setting, there are numerous unpredictable factors, and it is quite common for single kicks not to land. However, we often focus on studying kicks that do land, overlooking the fact that there are more kicks that miss the target in actual competition. In recent years, some scholars (Gulledge and Dapena, 2008; Xu et al., 2019; Vales-Alonso et al., 2023) have suggested that the main indicators of successful kicks are influenced by various factors, including the speed of the athlete’s kicking motion, the distance from the target, timing, psychological attributes, as well as the opponent’s stance, positioning, the competition environment, and lighting conditions. With the gradual improvement of Taekwondo rules and tactical systems, the occurrence of “missed” roundhouse kicks can negatively impact the effectiveness of kicking techniques (Falco et al., 2013), tactical planning (Ouergui et al., 2013), match outcomes (Ambroży et al., 2020), and even increase the risk of non-contact acute knee injuries (Jae-Ok and Voaklander, 2016; Jeong and Chun B, 2022). Therefore, reducing the incidence of “missed” roundhouse kicks is of fundamental importance. Currently, surface electromyography (sEMG) technology has become mature in the field of competitive sports. Both domestic and international scholars have utilized this technology to analyze the activity patterns and characteristics of surface EMG during Taekwondo techniques (Dong et al., 2013, Haifeng; Guo, 2013; Medina et al., 2022). In this context, the author employed sEMG technology to investigate the patterns, differences, and the extent of impact between “hit” and “miss” in roundhouse kicks. In this study, under laboratory conditions, high-speed cameras were used to capture the motion trajectories when athletes executed “hit” and “miss” targets with roundhouse kicks. Combining principles of human movement kinematics and dynamics with theoretical knowledge and practical experience, the research aimed to identify the similarities and differences between “hit” and “miss” in roundhouse kicks and assess their impact on movement speed. The goal is to provide theoretical insights to help Taekwondo athletes enhance their combat abilities, increase their movement speed, and improve kicking effectiveness.

## 2 Research object and research method

### 2.1 Research subjects

This study utilized G-Power software for a repeated measures analysis of variance to calculate the sample size. An effect size (d) of 0.4, significance level (A) of 0.05, and power of 0.9 were chosen, with six measurements per test. The analysis determined that a minimum of 10 participants is required. Therefore, this study focused on 10 outstanding female Taekwondo athletes from a specific province, all of whom possess national-level and above skills. The selection criteria for the participants were as follows: (1) The subjects had several years of professional team training experience and maintained an excellent level of athletic performance; (2) They had no sports injuries or other health issues and were not dependent on medication; (3) Within the 48 h preceding the tests, they had not undergone any strenuous training, and their physical fitness, competitive status, and athletic performance were normal; (4) The collected data from the tests were all valid. Prior to the testing, all participants provided informed consent by signing a voluntary agreement. The tests took place at the Sports Biomechanics Laboratory in the Sino-Russian Science and Technology Industrial Park located in the Yantai High-tech Zone, Shandong Province. Additionally, this research received approval from the Ethics Committee of Ludong University (Approval No: LDU-IRB202106011).

### 2.2 Testing instruments and materials

The tests were conducted using the EMG Noraxon Ultium 32 wireless surface electromyography (sEMG) testing equipment, the Vicon Nexus 1.85 (VICON^Ⓡ^, United Kingdom) from the United Kingdom for motion capture, and 12 high-definition cameras with 16-megapixel resolution each (Basler2.3.5, United Kingdom). Other testing equipment included disposable sEMG electrodes, medical cotton balls, computers, alcohol wipes, tissues, adhesive tape, razors, and subjects’ attire, among others.

### 2.3 Surface EMG signal acquisition and processing

The study selected the following seven pairs of major lower limb muscles for testing in the roundhouse kick (right leg kick) technique: left and right Erector Spinae, left and right Gluteus Maximus, left and right Rectus Femoris, left and right Biceps Femoralis, left and right Semitendinosus Semimembranosus, and left and right Gastrocnemius. The data was collected at a frequency of 2000 Hz. After testing, the data was imported into MATLAB, and time-domain metrics were calculated using the corresponding software.

### 2.4 Test method

① Instrument Setup: The laboratory testing personnel first start the 3D motion capture system, wireless surface electromyography (EMG Noraxon Ultium 32) testing equipment, the Vicon Nexus 1.85 (VICON^Ⓡ^, UK), and high-definition cameras. After verifying that everything is functioning correctly, calibration is performed. The testing environment is calibrated, and informed consent forms are distributed to the participants who then change into appropriate attire.

② Participant Preparation: All testing participants wear tight-fitting pants. The testing procedure, movements, and safety instructions are explained to them. Basic physical measurements of the participants are taken. Before the testing begins, participants engage in a brief 15-min warm-up session, including dynamic stretching and specific preparation exercises, to prevent sports injuries.

**FIGURE 1:**
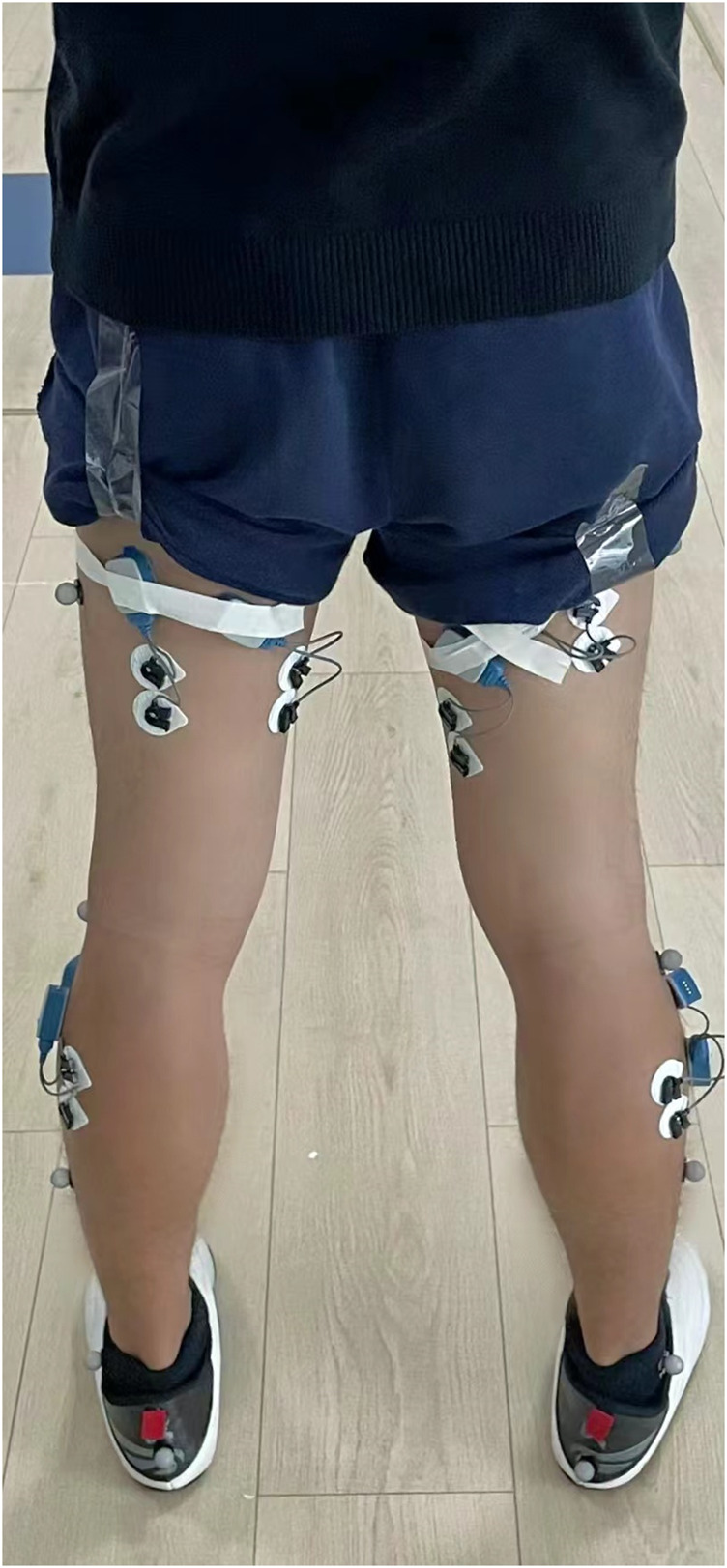
Diagram of electrode placement for athletes.

③ Placing Markers and Electromyography Electrodes: During the preparation process, one operator is responsible for attaching markers, while another provides assistance. Markers are attached to the subject’s hip, knee, and ankle joints. The area where markers are placed should be shaved to remove any hair, and the skin should be cleaned with alcohol to prepare the muscles for testing. After the alcohol has evaporated, the electrodes are positioned longitudinally (along the direction of muscle fibers) on the muscle (as shown in Figure 1) (Stegeman and Hermens H, 2007). They are then secured in place using skin tape and adhesive materials for static recording to establish a model.

**FIGURE 2:**
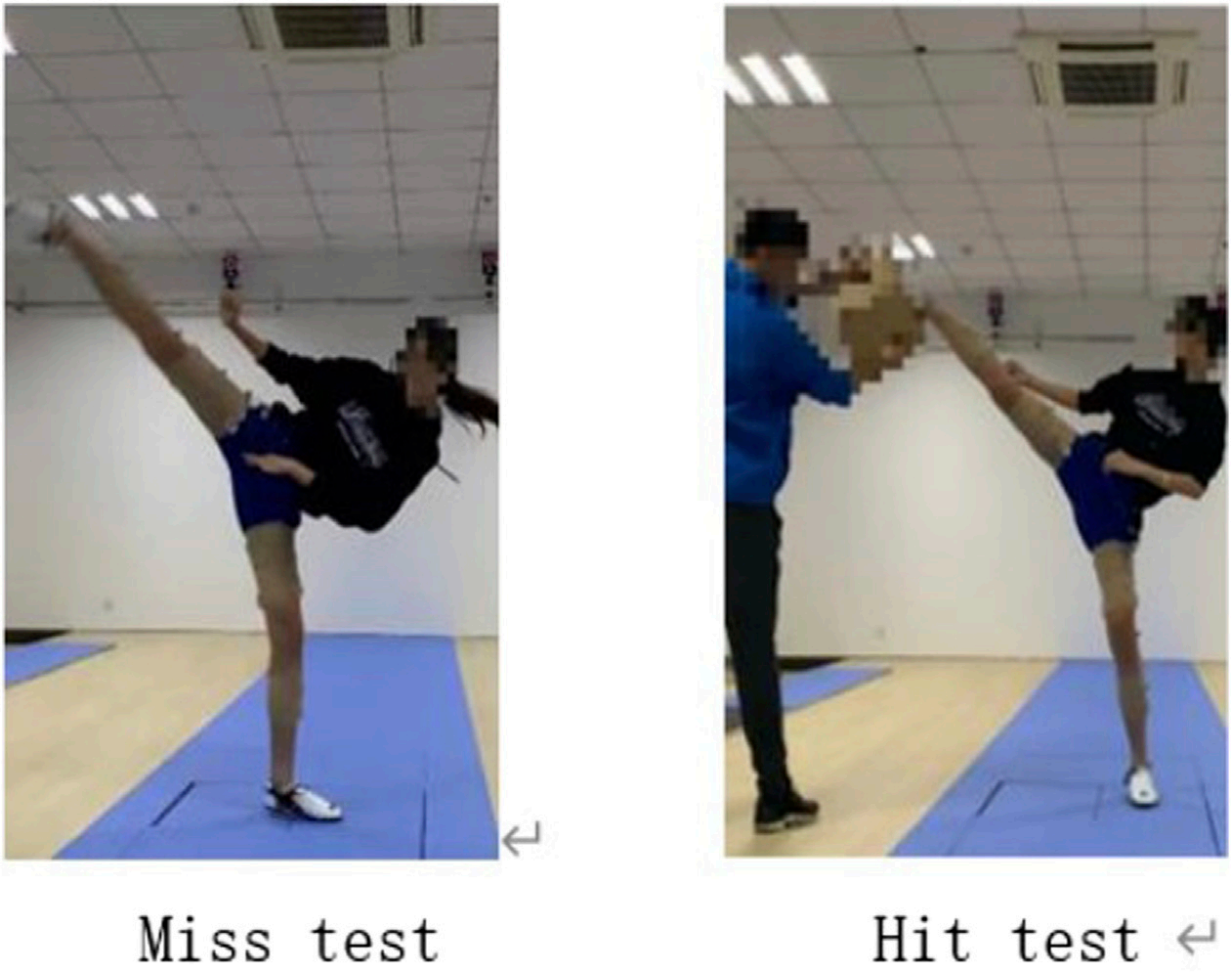
Schematic diagram of test action.

After the testing begins, participants follow verbal commands from the testing personnel. They use the roundhouse kick technique to rapidly strike a specified target positioned at the height of each participant’s head. An action in which the target is successfully struck is defined as a “hit.” In cases where the testing personnel unexpectedly withdraw the target during the initiation phase of the participant’s kick, and the participant fails to effectively strike the target, this action is defined as a “miss.” After each kicking action is completed, the researcher subjectively evaluates the performance and selects one excellent example of a “hit” and one example of a “miss” as the sample actions for this study (as shown in Figure 2) (Ouergui et al., 2013).

### 2.5 Action phase division

**FIGURE 3:**
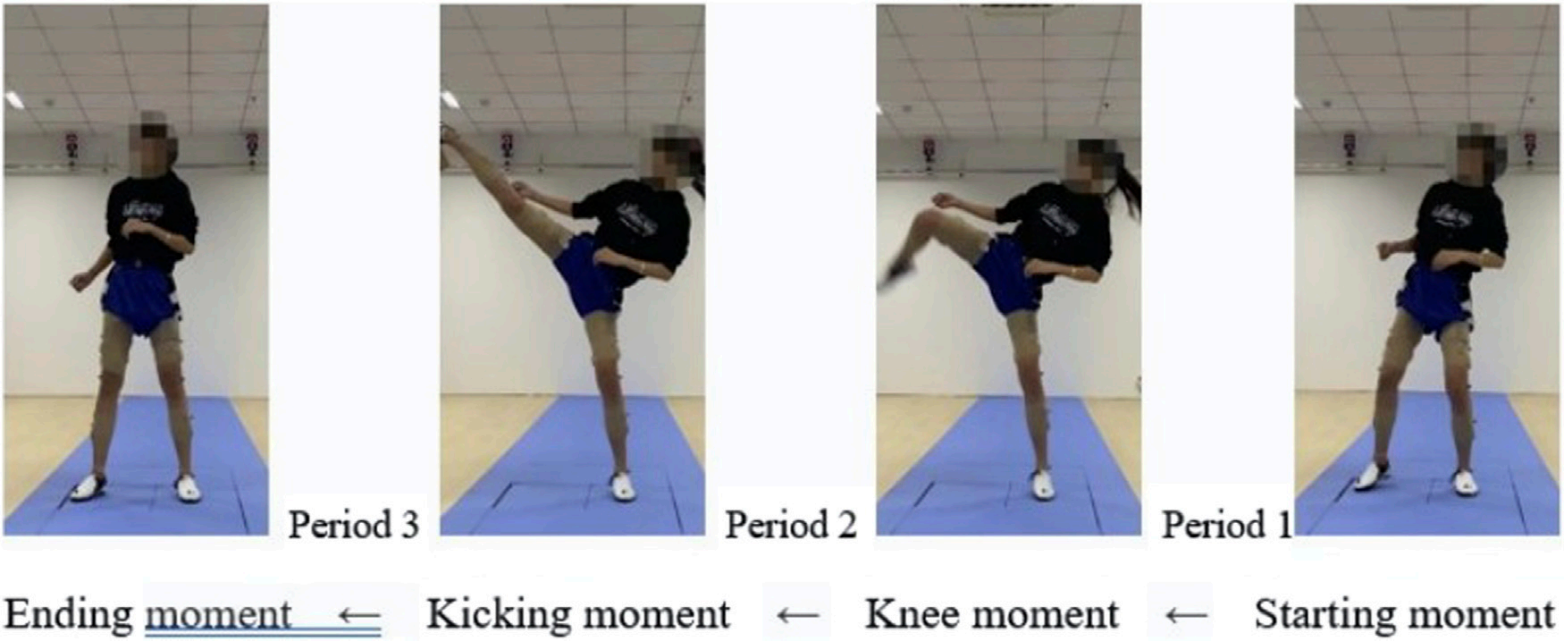
Stage division of the roundhouse kick action.

In accordance with the time-domain analysis of surface electromyographic signals, and considering the characteristics of the roundhouse kick technique, the testing action is divided into four “moments” and three “phases,” as shown in Figure 3. The moment when the swinging leg’s foot strikes the force platform is defined as the “initiation moment.” The “knee-up moment” is set when the swinging leg is raised, and the knee joint angle reaches its minimum. The “kicking moment” is set when the swinging leg fully hits the central target, and the knee joint angle is 180°. Finally, the moment when the swinging leg is retracted after the kick and touches the ground is defined as the “termination moment.” The intervals between these moments are defined as phases, with the period from the initiation moment’s end to the knee-up moment’s beginning designated as Phase 1 (Initiation Phase). The period from the knee-up moment’s end to the kicking moment’s beginning is designated as Phase 2 (Kicking Phase). Lastly, the period from the end of the kicking moment to the beginning of the termination moment is designated as Phase 3 (Recovery Phase) (Estevan et al., 2016).

### 2.6 Mathematical and statistical method

The raw electromyographic data obtained from the tests were imported into C3D format files. The Noraxon MR3.16 software, provided with the Noraxon32 system, was used to export the standard surface electromyographic reports, including Integrated EMG (IEMG) data. Statistical analysis was performed using SPSS 25.0. The differences between the “hit” and “miss” groups were analyzed using paired-sample *t*-tests. And then we performed a *post hoc* test, Electromyographic values are expressed as mean ± standard error. The significance level was indicated as follows: “*” for *p* < 0.1, “**” for *p* < 0.05, and “***” for *p* < 0.01.

## 3 Results

### 3.1 Activation timing of muscles in roundhouse kick “miss” and “hit” actions

The researchers analyzed the muscle activation sequence in high-level athletes performing the front leg roundhouse kick, comparing “hit” and “miss” techniques. They defined muscle activation as when the amplitude exceeded the baseline level by 30% three times within a 100 Hz cycle (Cao et al., 2021).

**FIGURE 4:**
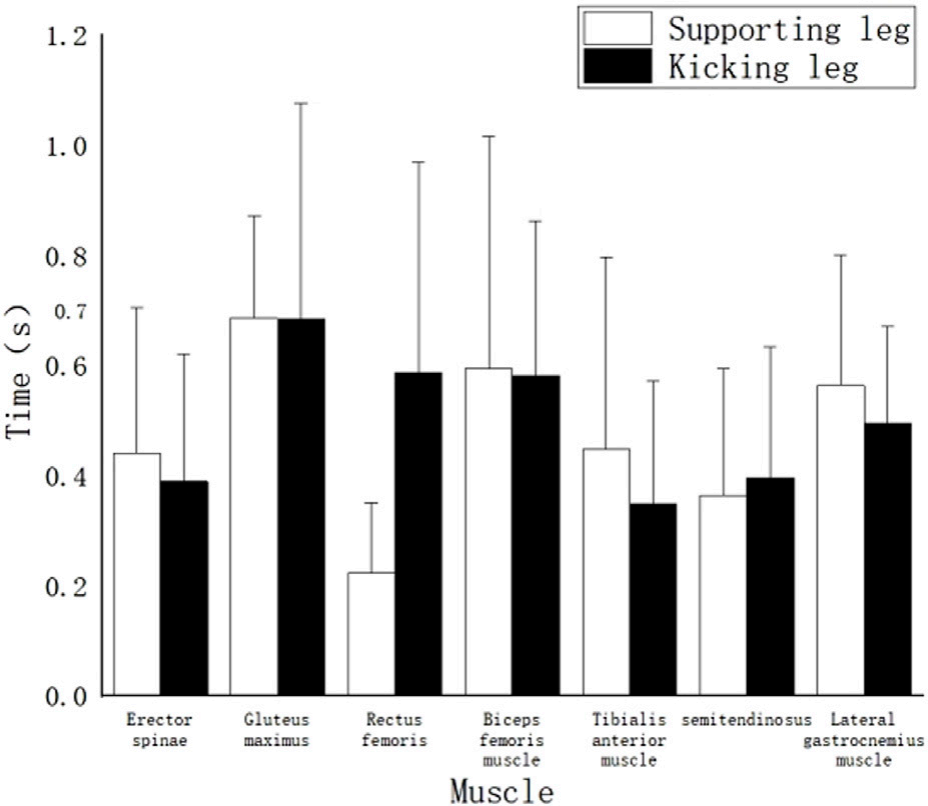
Timing of muscle activation of the “hit” technique of the roundhouse kick.

In Figure 4, which illustrates the muscle activation sequence for the front leg roundhouse kick “hit” technique in high-level athletes, the following sequence of muscle activation was observed:Initially activated muscle: Supporting-side Rectus Femoris (0.22s). Subsequently activated muscles, close in activation time: Swing-side Anterior Tibialis (0.35s), Semitendinosus Semimembranosus (0.39s), Erector Spinae (0.39s), and supporting-side Semitendinosus Semimembranosus (0.36s). Muscles activated next: Supporting-side Erector Spinae (0.44s), Anterior Tibialis (0.45s), and Swing-side Gastrocnemius (0.49s). Following those, supporting-side Gastrocnemius (0.56s), Swing-side Biceps Femoralis (0.58s), supporting-side Biceps Femoralis (0.59s), and Swing-side Rectus Femoris (0.59s) were activated. Their activation times were close. The last muscle to be activated was Gluteus Maximus, with activation times for both supporting and swing sides at 0.68s.

It is important to note that all of the muscle activation times mentioned above occurred prior to the moment of kicking.

**FIGURE 5:**
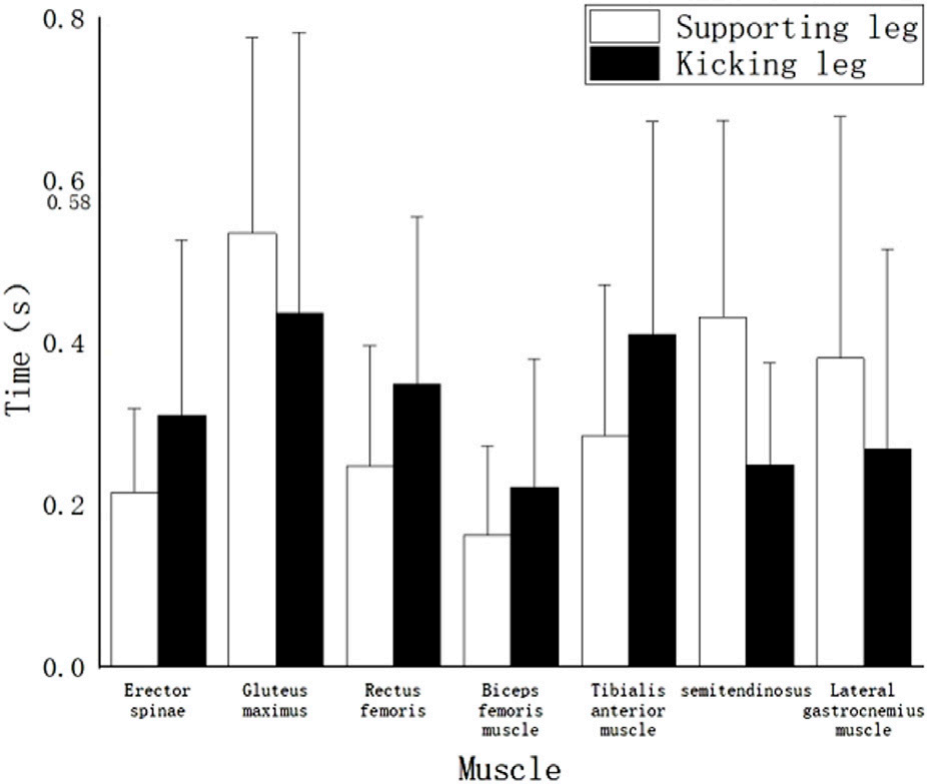
Muscle activation timing of the “miss” technique of the roundhouse kick.

Using the knee joint angle of 180° during the swing leg as a reference point (1.1 m), Figure 5 illustrates the muscle activation sequence for the front leg roundhouse kick “miss” technique. The results reveal the following sequence of muscle activations: The initial muscles to activate were the left and right Biceps Femoralis at 0.16s and 0.22s, respectively. Subsequently, the left Rectus Femoris (0.25s), left Erector Spinae (0.22s), left Anterior Tibialis (0.28s), right Semitendinosus Semimembranosus (0.25s), and right Gastrocnemius (0.27s) muscles were activated in quick succession. These were a densely activated group of muscles with relatively early activation times. Following this group, the right Erector Spinae (0.31s), right Rectus Femoris (0.35s), left Gastrocnemius lateral head (0.38s), right Anterior Tibialis (0.41s), left Semitendinosus Semimembranosus (0.43s), and right Gluteus Maximus (0.44s) muscles were activated. The last muscle to be activated was the left Gluteus Maximus at 0.53s.

It is important to note that the activation times for all of these muscles occurred early in relation to the defined time point.

### 3.2 Work timing of muscles in roundhouse kick “miss” and “hit” techniques

**FIGURE 6:**
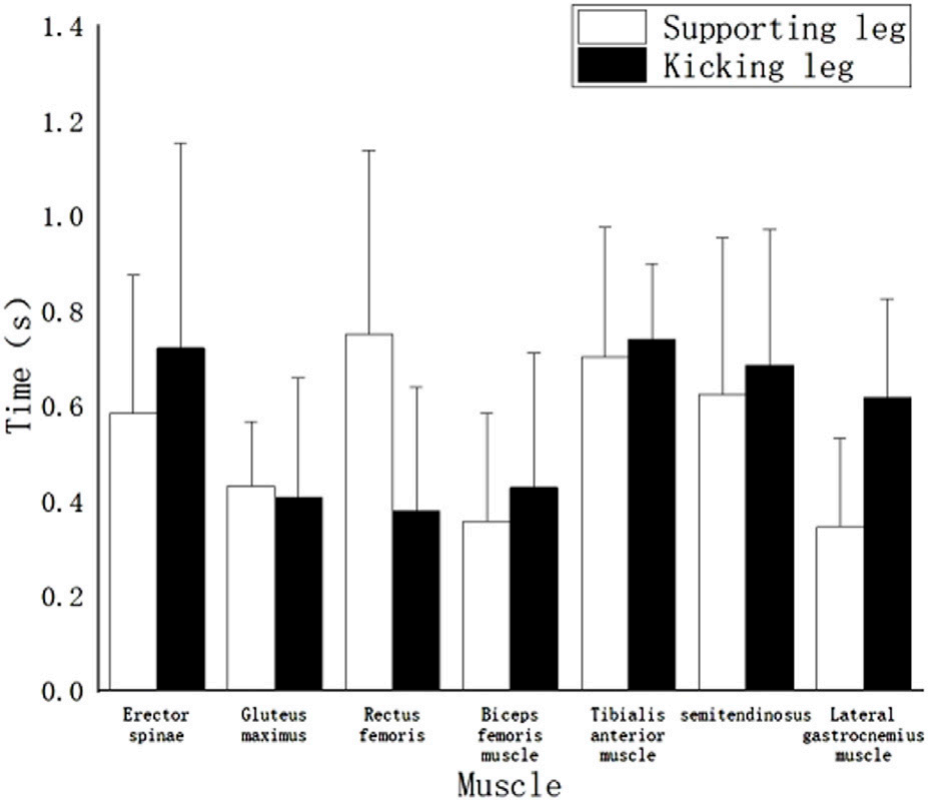
The working time of each muscle of the “hit” technique action.

Figure 6 displays the duration of muscle activity in high-level athletes during the “hit” technique of the roundhouse kick. The results reveal the following: The muscle with the longest duration of activity is the left Rectus Femoris (0.75s). Following closely are the right Anterior Tibialis (0.74s), right Erector Spinae (0.72s), left Anterior Tibialis (0.70s), and right Semitendinosus Semimembranosus (0.68s). These muscles form a group with longer working durations. The right Gastrocnemius lateral head (0.62s), left Semitendinosus Semimembranosus (0.62s), and left Erector Spinae (0.58s) have moderate working durations. Muscles with relatively shorter working durations include the left Gluteus Maximus (0.43s), right Biceps Femoralis (0.43s), right Gluteus Maximus (0.40s), right Rectus Femoris (0.38s), and left Biceps Femoralis (0.36s). Notably, the left Gastrocnemius lateral head has the shortest working duration, lasting only 0.34s.

**FIGURE 7:**
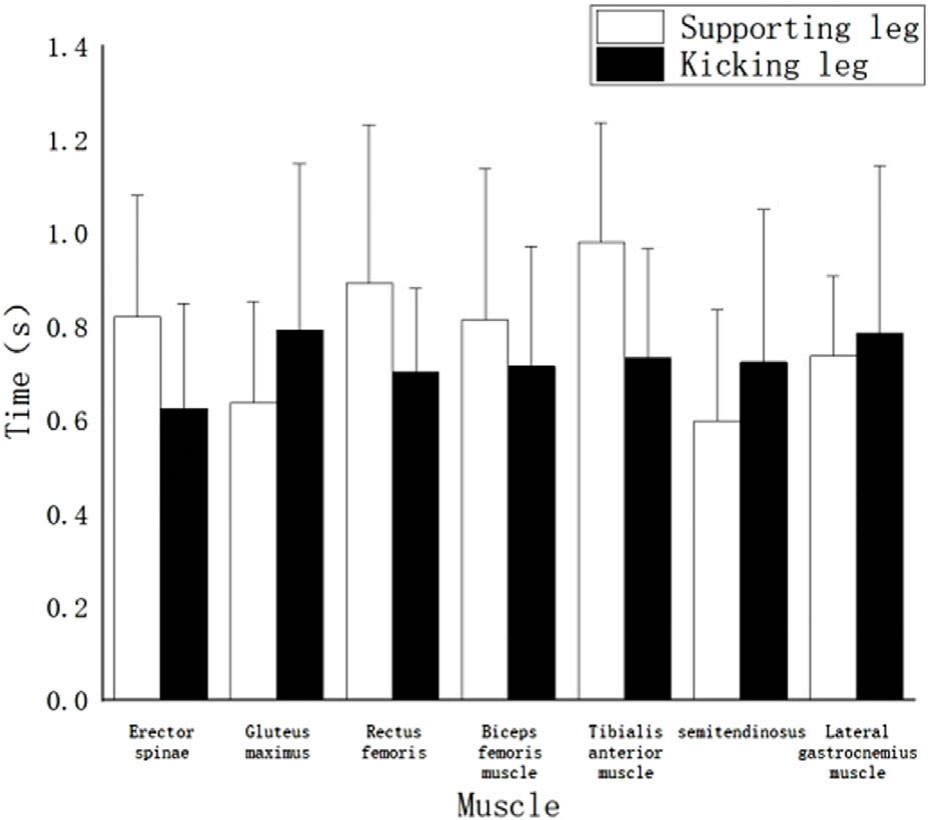
The working time of each muscle of the “miss” technique.

Figure 7 presents the duration of muscle activity during the “miss” technique of the roundhouse kick. The results indicate the following: The muscle with the longest duration of activity is the left Anterior Tibialis (0.95s). Following closely are the left Rectus Femoris (0.88s), left Erector Spinae (0.82s), left Biceps Femoralis (0.79s), right Gastrocnemius lateral head (0.77s), right Gluteus Maximus (0.76s), right Anterior Tibialis (0.73s), right Biceps Femoralis (0.72s), left Gastrocnemius lateral head (0.71s), right Semitendinosus Semimembranosus (0.7s), right Rectus Femoris (0.68s), left Gluteus Maximus (0.62s), and right Erector Spinae (0.62s). The muscle with the shortest duration of activity is the left Semitendinosus Semimembranosus (0.58s).

### 3.3 Comparison of electromyographic activity in the “miss” and “hit” techniques at different time intervals

In order to compare the differences between “miss” and “hit” techniques, the researchers conducted a comparison of the integrated electromyographic (IEMG) indicators for the 14 muscles in each of the three time periods for both “miss” and “hit” techniques. This analysis helps reflect the different muscle engagement patterns between “hit” and “miss” techniques. Tables 2 display the statistical analysis results of the IEMG values for the 14 muscles in each time period for both “miss” and “hit” techniques.

By comparing the two techniques, it was observed that during Time Period 3, several muscles showed varying degrees of differences in IEMG values. The left Gluteus Maximus, right Gluteus Maximus, right Rectus Femoris, and left Anterior Tibialis had relatively significant differences (*p* < 0.1). The left Gastrocnemius, left Rectus Femoris, right Anterior Tibialis, and right Biceps Femoralis exhibited significant differences (*p* < 0.05). The right Semitendinosus Semimembranosus displayed highly significant differences (*p* < 0.01) in IEMG values. No significant differences were found in the other muscles. In addition to this, we performed a *post hoc* test, which was highly similar to the results of the paired samples *t*-test above. The left lateral gastrocnemius *post hoc* test showed F = 8.590, *p* = 0.013. The left rectus femoris *post hoc* test showed F = 10.847, *p* = 0.006. The right tibialis anterior *post hoc* test showed F = 5.411, *p* = 0.038. The right biceps femoris *post hoc* test showed F = 10.478, *p* = 0.007. The right biceps femoris *post hoc* test showed F = 10.478, *p* = 0.007. The right biceps femoris *post hoc* test showed F = 10.478, *p* = 0.007. Post-hoc test of the right semitendinosus muscle showed F = 9.069, *p* = 0.011.

**FIGURE 8:**
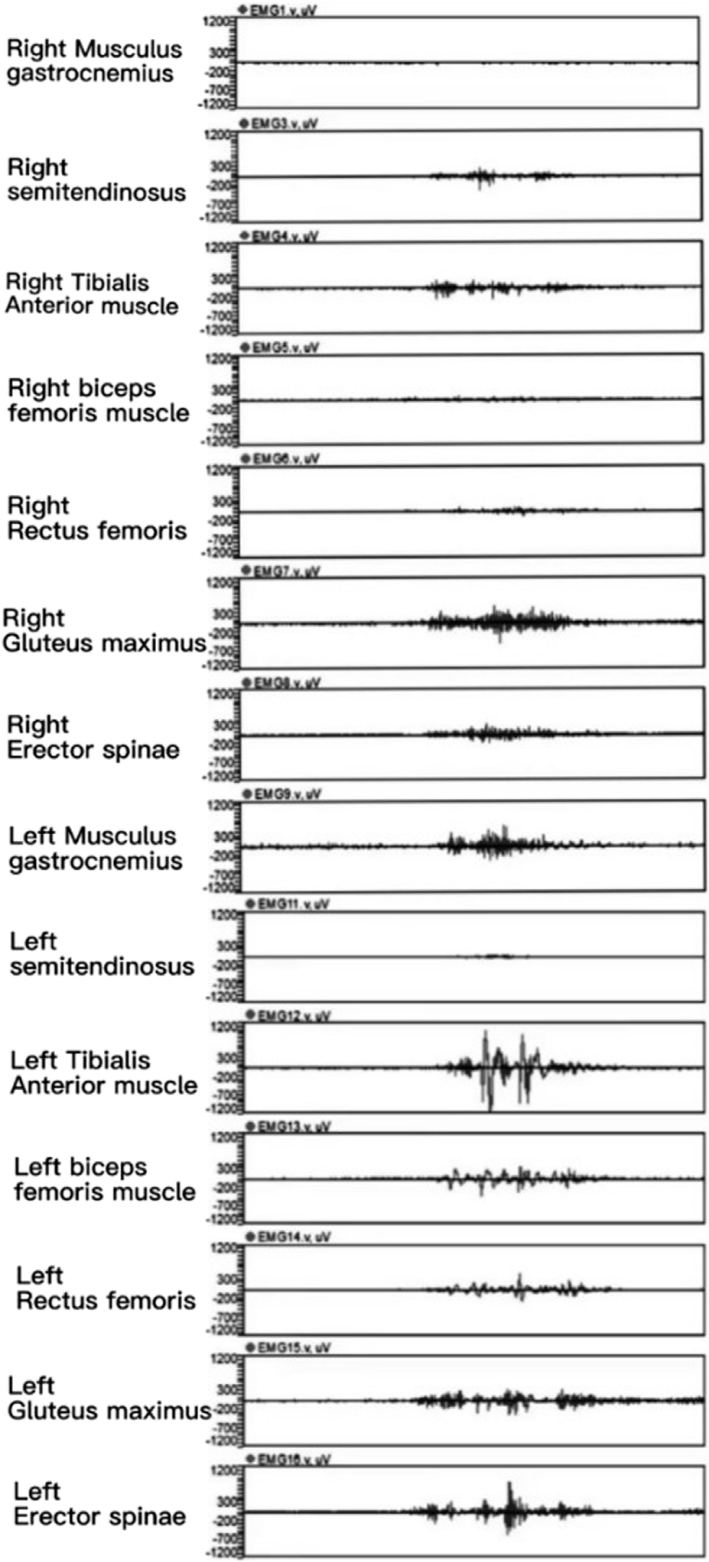
A schematic diagram of an EMG from one of the participating athletes.

Figure 8 displays the original electromyographic signals collected from a representative athlete during a roundhouse kick. It is evident that the Erector Spinae, Gluteus Maximus, Semitendinosus Semimembranosus, and Gastrocnemius muscles on the swing side exhibited varying degrees of higher amplitudes compared to the supporting side, and they had longer durations.

## 4 Discussion

### 4.1 Surface electromyographic characteristics of the lead leg roundhouse kick “miss” and “hit” actions

#### 4.1.1 Analysis of muscle activation in roundhouse kick “miss” and “hit” actions

As shown in Figures 4, 5, taking the point at which the kicking leg extends to 180° as a time reference, it is observed that both “miss” and “hit” roundhouse kick techniques in this study, which involve high kicks, have relatively late muscle activation times compared to the time reference. However, for both techniques, the activation of all muscles occurs to varying degrees before the time reference. There are also differences in the sequence of muscle activation between the two techniques:

For the “hit” technique, the muscle activation sequence of the swinging leg is as follows: Anterior Tibialis → Erector Spinae → Semitendinosus Semimembranosus → Gastrocnemius (lateral head) → Biceps Femoralis → Rectus Femoris → Gluteus Maximus.

For the “miss” technique, the muscle activation sequence of the swinging leg is as follows: Biceps Femoralis → Semitendinosus Semimembranosus → Gastrocnemius (lateral head) → Erector Spinae → Rectus Femoris → Anterior Tibialis → Gluteus Maximus.

The roundhouse kick is characterized by a combination of horizontal and vertical movement toward the target by the athlete. This movement involves rapid forward rotation of the pelvic axis, hip joint abduction, hip joint flexion, and knee joint extension (Kim et al., 2010). Kicking speed places high demands on the hip flexors and adductors, and well-developed hip joint muscles are essential for optimal activation during kicking, especially in techniques involving instep kicks (such as soccer shots and kickboxing roundhouse kicks) (Brophy et al., 2007).

It is interesting to note that while the final muscle activated in both techniques is the same (the gluteus maximus), the initial muscle activation differs significantly. In the “hit” technique, the tibialis anterior muscle is activated first, whereas in the “miss” technique, the biceps femoris muscle is activated initially. This difference may be attributed to the rich practical experience of professional athletes. The observation that athletes tend to perform the “miss” technique more in line with the standard technique, particularly during the knee-raising phase, indicates their utilization of core abdominal strength to control hip rotation to a position that is closer to horizontal and vertical to the ground (Ervilha et al., 2020). This position is maintained until the entire leg is fully folded and ready for the recoil phase. However, this phase necessitates the initiation of certain thigh muscles (Thibordee and Prasartwuth, 2014). Consequently, the muscles activated first in the “miss” technique are the biceps femoris and semitendinosus semimembranosus, with muscle activation times closely aligned. The muscles of the lower leg activate more slowly, which suggests that the thigh muscles are driving the lower leg muscles. In contrast, the “hit” technique often differs due to extensive practical combat experience, target training, and protective gear training. To ensure the effectiveness of the attack, athletes tend to not complete the knee-raising phase fully. As a result, the thigh muscles remain unfolded to a minimal angle, or the knee joint angle exceeds 60°, leading to a direct leg recoil. This observation explains why the rectus femoris muscle is the muscle activated first in the “hit” technique, as this technique follows a sequence where the lower leg muscles initiate the activation of the thigh muscles (Aandahl et al., 2018a). The observation that most muscles in the supporting leg are activated earlier than those in the swinging leg for both techniques implies that in the roundhouse kick, the muscles of the supporting leg initiate the force by pushing off the ground, with the left side muscles following suit. During the phase from raising the knee to recoiling, athletes are in an unstable state when they rely on single-leg support. The relationship between muscle activity in stable moments (preparation and completion) and unstable states follows a U-shaped curve (Dong and Yuan, 2019), and the transition between stable and unstable states is influenced by a combination of biological and dominant factors (Kim et al., 2017).

#### 4.1.2 Muscle work timing in roundhouse kick “miss” and “hit” actions

The duration of muscle activity to some extent reflects the level of contribution of the corresponding muscles to the completion of the action. As shown in Figure 6, in the “hit” technique, the left and right Rectus Femoris muscles have the longest working time, with the right Rectus Femoris muscle being particularly prominent, covering almost the entire duration of the “hit” action. It can be considered the core muscle for this action. Following this, the left Biceps Femoralis, left Rectus Femoris, right Gastrocnemius (lateral head), right Gluteus Maximus, and left Erector Spinae muscles also have relatively late activation times but continue to work until the end of the action, making them important muscles for this technique.

It is worth mentioning that the right Biceps Femoralis, with the shortest working time among the muscles, works for only 0.79 m. This reflects that the right Biceps Femoralis is not significantly involved in the “hit” action from the kicking phase to the end, suggesting that its contribution to this action is minimal.

Unlike the “hit” technique, in the “miss” technique, all the muscles have working durations that extend until the end of the action. The muscles with the longest working durations in this technique are the left Biceps Femoralis, left Anterior Tibialis, left Rectus Femoris, and left Gluteus Maximus, all of which are muscles of the supporting leg. These muscles play an essential role in this action. Except for the Semitendinosus Semimembranosus and Gastrocnemius muscles, all the muscles of the supporting leg have working durations longer than those of the kicking leg. The working duration of the swinging leg muscles is directly proportional to their activation time, with the muscles that are activated first having the longest working durations.

Through a comparison of the two techniques, it was found that the time period for the “hit” action is significantly shorter than that for the “miss” technique (Su et al., 2019). The author believes that this is because in the “hit” technique, athletes, after striking the target, experience a reactive force from the target on their instep, allowing the kicking leg to actively fold and retract promptly after hitting the target. In contrast, in the “miss” action, due to the unpredictability of when the target is withdrawn, the kicking leg fails to make contact with an object to generate a reactive force, preventing athletes from completing the “retraction of the leg” phase of the roundhouse kick and forcing the kicking leg to land directly.

#### 4.1.3 Analysis of muscle work in roundhouse kick “miss” and “hit” actions

Electromyographic values can directly reflect the force exerted by the corresponding muscles (Merletti and Lo Conte, 1995). As shown in Tables 1, 2, during time period 1, there were no statistically significant differences in the integrated electromyographic values of muscles between the “miss” and “hit” actions (*p* > 0.05). In the “miss” technique, the muscle with the highest electromyographic activity during this period was the left erector spinae muscle, and its activity significantly exceeded that of other muscles. This observation clearly reflects that athletes rely heavily on the left erector spinae muscle for support during the initiation phase of the “miss” action. Other muscles had relatively lower and uniform electromyographic activity, with some exceptions. Overall, the electromyographic activity on the supporting side was higher to varying degrees than that on the kicking side.

**TABLE 1:** Participant demographic information table.

Number	Gender	Age	Weight (kg)	Height (cm)	Skill level	Years of training
Subject 1	Female	24	65	177	Elite Athlete	9
Subject 2	Female	25	62	168	Level 1	6
Subject 3	Female	22	78	180	Level 1	8
Subject 4	Female	26	62	176	Level 1	10
Subject 5	Female	22	72	178	Level 1	8
Subject 6	Female	20	60	168	Level 1	6
Subject 7	Female	24	78	182	Level 1	8
Subject 8	Female	27	78	188	Level 1	8
Subject 9	Female	28	78	172	Level 1	8
Subject 10	Female	26	78	179	Level 1	8

**TABLE 2:** Comparison of integrated electromyography differences of 14 muscles during the “hit” and “miss” actions of the roundhouse kicsk at different time periods.

Muscles	Hit integral EMG value/(μV·s)					Miss integral EMG value/(μV·s)				
	Phase 1		Phase 2		Phase 3	Phase 1		Phase 2		Phase 3
Right Musculus gastrocnemius	2.20 ± 0.6	2.8 ± 0.7		28.89 ± 4.7		1.67 ± 0.2	1.93 ± 0.3		23.21 ± 4.9	
Left Musculus gastrocnemius	1.17 ± 0.6	1.6 ± 0.6		28.59 ± 6.0**		2.74 ± 0.3	2.15 ± 0.5		8.28 ± 2.7**	
Right semitendinosus	1.22 ± 0.1	3.7 ± 0.9		29.97 ± 3.9***		3.45 ± 1.1	2.77 ± 0.6		14.51 ± 3.9***	
Left semitendinosus	1.7 ± 0.4	3.5 ± 1.3		8.7 ± 2.4		2.14 ± 1.2	3.07 ± 0.7		8.86 ± 2.1	
Right Tibialis Anterior muscle	1.26 ± 0.1	2.6 ± 0.8		28.2 ± 2.3**		1.21 ± 0.1	1.65 ± 0.4		16.52 ± 4.7**	
Left Tibialis Anterior muscle	1.41 ± 0.4	5.1 ± 0.2		51 ± 7.8*		1.56 ± 0.3	3.71 ± 0.5		28.42 ± 5.1*	
Right biceps femoris muscle	1.78 ± 0.4	1.63 ± 0.6		20.9 ± 4.3**		2.36 ± 0.6	2.23 ± 0.3		8.63 ± 1.6**	
Left biceps femoris muscle	1.96 ± 0.9	1.9 ± 0.8		11.12 ± 3.3		1.74 ± 0.5	1.11 ± 0.3		4.32 ± 0.3	
Right Rectus femoris	1.12 ± 0.2	1.87 ± 0.7		44.5 ± 12.6*		1.36 ± 0.3	1.54 ± 0.5		18.76 ± 9.2*	
Left Rectus femoris	0.85 ± 0.4	1.5 ± 0.5		33.5 ± 6.6**		0.9 ± 0.2	1.31 ± 0.3		9.94 ± 2.6**	
Right Gluteus maximus	1.21 ± 0.4	1.7 ± 0.4		17.3 ± 4.7*		1.68 ± 0.4	1.93 ± 0.5		7.8 ± 2.1*	
Left	0.96 ± 0.3	1.2 ± 0.6		39.2 ± 9.2*		1.28 ± 0.3	1.45 ± 0.3		16.12 ± 2.6*	
Gluteus maximus										
Right	2.46 ± 0.5	3.0 ± 0.9		59.5 ± 30.7		2.98 ± 1.7	2.59 ± 0.9		121.64 ± 43.5	
Erector spinae										
Left	6.4 ± 3.3	11.9 ± 4.9		73.4 ± 32.8		1.75 ± 0.4	3.06 ± 1.2		32.56 ± 11.8	
Erector spinae										

Note: Integral electromyographic values are expressed as x ± sd (mean ± standard error). Paired samples *t*-test was conducted for the same muscles in the “hit” and “miss” categories in the table, where * indicates *p* < 0.1, ** indicates *p* < 0.05, *** indicates *p* < 0.01.

In the “hit” action during this period, the muscles with the highest activity were the semitendinosus, erector spinae, biceps femoris short head, and biceps femoris long head. Notably, the supporting side muscles such as the semitendinosus, erector spinae, and biceps femoris long head, as well as the swinging side’s biceps femoris short head, exhibited significantly higher muscle activity than their counterparts on the opposite side. These muscles play a crucial role in the initiation phase of the front leg roundhouse kick “hit” technique. The muscles with the least work in both techniques were the rectus femoris, which had the least contribution to the initiation phase.

During this period, all muscles were in an activated or pre-activated state. Both “miss” and “hit” techniques had relatively low overall electromyographic activity, as the supporting leg’s ground push and the kicking leg’s initiation occurred simultaneously. The folding angle of the kicking leg and the height of the knee lift determined the striking height and power (Aandahl et al., 2018b).

During time period 2, in both the “miss” and “hit” actions, the striking height is similar. The primary muscles involved in both actions include the bilateral erector spinae muscles, semitendinosus, and tibialis anterior. Furthermore, in the swinging side, the semitendinosus, gluteus maximus, and rectus femoris had integrated electromyographic values higher than those on the supporting side. This indicates that after the phase of muscle pre-activation, the muscles responsible for hip, knee, and ankle flexion and extension in the swinging leg are the primary force-producing muscles during this time period (Wąsik and Shan, 2015).

Based on the data from Tables 1, 2, it is observed that in time period 3, the left biceps femoris short head, right semitendinosus, right tibialis anterior, left tibialis anterior, right rectus femoris, right vastus lateralis, left vastus lateralis, right gluteus maximus, and left gluteus maximus exhibit significant differences between the “miss” and “hit” techniques. In these muscles, the integrated electromyographic values for the “miss” technique are higher to varying degrees compared to the “hit” technique, with the difference being most significant in the right semitendinosus (*p* < 0.01). Other muscles without significant differences are primarily on the supporting side, indicating that, apart from the moment of retraction, the supporting side muscles do not significantly differ in work and force production between the two techniques.

In this time period, the muscles with the highest work in the “miss” action are the erector spinae on both sides, vastus lateralis on both sides, rectus femoris on both sides, tibialis anterior on both sides, biceps femoris short head on the right side, and biceps femoris long head on the right side. For the “hit” action, the muscles with the highest work are the erector spinae on both sides, rectus femoris on both sides, right vastus lateralis, right biceps femoris short head, and right tibialis anterior. These muscles are considered the core muscles for this time period. It is clear that in the “miss” action, the integrated electromyographic values for various muscle groups are greater than in the “hit” action.

During the stages from the kicking phase to the retraction phase, the dominant muscle group for hip joint is the hip flexor (rectus femoris) during the preparation to knee lift phases. Subsequently, the hip extensors (biceps femoris long head and semitendinosus) take over as the dominant muscle group until the end of the kicking action. In contrast, the knee extensors (hamstrings) become the dominant muscle group from stage 1 (two-thirds of the entire action) onwards (Xu et al., 2020; Aandahl et al., 2018a; Miziara et al., 2019). The author believes that in the “miss” action, since there is no target to strike, the athlete’s core stability is somewhat compromised. This results in the kicking leg not being able to retract actively and landing smoothly. During this phase, various muscle groups need to continue generating force to control the inertia of the whipping action, leading to an extended action cycle and a slow landing of the kicking leg. This is also the main reason why the integrated electromyographic values of the erector spinae on the supporting side are greater than those on the swinging side.

Tables 1, 2 indicate that the integrated electromyographic values for various muscles are, to varying degrees, greater in the “miss” action compared to the “hit” action. Although the integrated electromyographic values are lower in the “hit” action, the action cycle is shorter, and the kicking leg retracts rapidly. The integrated electromyographic values for core muscles in the “hit” action are greater than those on the supporting leg, which is consistent with the results of HWANG (1986).

### 4.2 Training insights

#### 4.2.1 Competition rules trends

Since the introduction of electronic protective gear in world-class Taekwondo competitions, the World Taekwondo Federation (WTF) has made frequent rule modifications, gradually refining the high-tech fair competition environment system that records points using electronic protective gear and headgear. At each Olympic Games, they analyze rule deficiencies and make corrections. Starting from the London Olympics, they strictly enforced penalties for “back-avoidance” violations that affected the indomitable warrior spirit of Taekwondo. Today, they have strict penalties for stalling and negative behaviors that go against the spirit of sportsmanship, encouraging athletes to be more proactive (Zhang and Guan, 2017).

#### 4.2.2 Real-world technical and tactical use

Due to rule-oriented considerations, counterattacks have gradually decreased in world competitions, especially in middle and lower weight classes. In high-level competitions, the proportion of proactive attacking techniques is much higher than that of counterattacks and defensive techniques. The front leg roundhouse kick described in this paper is a counterattacking technique primarily used when the opponent initiates an attack after a defensive lapse. It involves kicking in response to the opponent’s attacking motion (Qiao and Li, 2016). Because this technique is a high-level action that places less demand on power when striking electronic headgear compared to protective gear, it is less predictable for the opponent. Although it may not be used frequently, its scoring rate is remarkably high, making it an effective tactical surprise. Additionally, in overtime periods, the scoring rate for counterattacks becomes even more prominent. In conclusion, the judicious use of counterattacking techniques is crucial for athletes to steer the direction of the game in their favor.

#### 4.2.3 Sports training orientation

In combat sports, it is often believed that a higher speed of execution leads to a higher scoring rate. However, the “miss” technique has a notably slower execution time compared to the “hit” technique. Both techniques involve a process where various leg muscle groups are lengthened and then rapidly contracted to generate force. These two components are critical factors determining the speed of these techniques. In the “miss” technique, the muscle contraction time during the recoiling phase is excessively long, leading to a slow execution. Therefore, whether in real combat situations or competitions, it is crucial to avoid the occurrence of the “miss” technique.

To address this issue, training should not only focus on the leg muscles but also pay attention to the training of the pelvic joint and associated muscles. In specialized physical training programs, the human body should be trained to utilize the small muscles of the lower leg to activate various muscle groups in the thigh through a lever system, thereby creating maximum force in the muscles and levers that are activated next in the kinetic chain. Furthermore, emphasis should be placed on the ability of muscles to contract maximally within the shortest time frame when in a relaxed state (Shi, 2013; Wang, 2013).

## 5 Conclusion

These two techniques exhibit differences in muscle activation times: in the “miss” technique, the thigh muscles drive the force in the leg, while in the “hit” technique, the leg muscles drive the force in the thigh. Additionally, there are disparities in the muscle work duration between these two techniques, with the “hit” technique having a shorter duration compared to the “miss” technique. In the “miss” technique, all muscles continue to work until the completion of the movement. This reflects the fact that the “miss” technique has a longer time duration, which should be avoided in real combat or competitions (Cho et al., 2021). During the first and second phases, there are no significant differences in muscle integrated electromyography values for both techniques. However, the integrated electromyography values are generally smaller. In the third phase, there are significant differences in the muscle work for both techniques. Notably, the “miss” technique shows higher integrated electromyography values for most muscles compared to the “hit” technique (Falco et al., 2009). This difference may be attributed to the longer time duration of the “miss” technique, among other factors (Valdes-Badilla et al., 2018). In both techniques, the muscles exhibit their maximum work during the third phase, indicating that the force applied during the recoiling phase is greater than during the kicking phase. The “miss” technique involves a larger range of rotational motion in the lower limb joints, which can lead to a less balanced body center of gravity and a greater impact on the speed of the technique (Yilmaz et al., 2021). On the other hand, the “hit” technique typically has a faster speed, and the movement chain of the body has less influence on the speed of the technique. It is recommended that coaches, during training, consider factors such as the opponent’s stance and position, the training environment, lighting conditions, and more (Casolinoet al., 2012). However, they should also take into account the specific characteristics of the roundhouse kick technique and incorporate footwork in various directions with the technique to enhance the quality of the strike. During training, it is important to focus on strengthening the muscles of the hip flexors and knee extensors when kicking, emphasizing the core muscles’ activation (Kim et al., 2011). For lateral support, coordination of muscle engagement and the rhythm of movement are crucial (Moreira et al., 2016). Additionally, simulating resistance in the initial stages of training can significantly improve the success rate of the “hit.” (Khazaei et al., 2023). Starting from practical combat situations during training, attention should be given to rules and the development trends of competitive tactics, allowing for a rational arrangement of tactical application and the updating of the sports training system.

## 6 Limitations of the study

Firstly, there are individual differences and skill levels; individuals may exhibit significant differences in skill levels and movement styles during the kicking process. These individual variations could have a certain impact on the interpretation and generalization of experimental results. Secondly, the participants’ performance in the experiment may be influenced by psychological factors, potentially leading to an incomplete simulation of kicking behavior. Finally, there are limitations in simulating the experimental environment due to constraints in the experimental site and equipment. The lack of a realistic environment may result in observed kinematic differences in the experiment compared to actual kicking scenarios. In addition, the sample size of the study (*n* = 10) is not enough to ensure the reliability of the study statistically, and such studies can be further explored in combination with kinematics in the future.

## Data Availability

The original contributions presented in the study are included in the article/supplementary material, further inquiries can be directed to the corresponding author.
